# Safety and Accuracy of Guided Interradicular Miniscrew Insertion: A Systematic Review and Meta-Analysis

**DOI:** 10.3390/jcm13247697

**Published:** 2024-12-17

**Authors:** Margalida Santmartí-Oliver, Adrià Jorba-García, Tania Moya-Martínez, Cristina de-la-Rosa-Gay, Octavi Camps-Font

**Affiliations:** 1 Faculty of Dentistry, Complutense University of Madrid, 28040 Madrid, Spain; msantmar@ucm.es; 2 Faculty of Medicine and Health Sciences, University of Barcelona, 08907 Barcelona, Spain; taniamoyamartinez01@gmail.com (T.M.-M.); crosagay@ub.edu (C.d.-l.-R.-G.); 3 IDIBELL Institute, 08908 Barcelona, Spain

**Keywords:** miniscrews, mini-implants, temporary anchorage, orthodontic anchorage devices

## Abstract

**Background**: Achieving ideal anchorage is crucial in orthodontics for controlled tooth movement. Miniscrews (MSs) have improved skeletal anchorage, but freehand placement poses risks like root damage and limited precision. Guided techniques, including radiographic guides and computer-assisted methods (static [sCAS] and dynamic [dCAS]), were developed to enhance accuracy and safety. **Objective**: This systematic review and meta-analysis aimed to evaluate the safety and accuracy of MS placement using different guidance approaches. **Materials**: A systematic search up to March 2024 identified studies on guided MS insertion, assessing safety (root contact/damage) and accuracy (angular, coronal, and apical deviations) of guided vs. freehand placement. Two reviewers assessed the risk of bias and study quality using RoB 2 for RCTs, NOS for cohort studies, and an adapted tool for pre-clinical studies. Random-effects meta-analysis was performed for studies with common parameters, and safety outcomes were pooled using logit-transformed proportions. Heterogeneity was evaluated with I² and χ² tests. **Results**: Eleven studies (652 MSs) were included, though no dCAS studies were analyzed. The only RCT had “some concerns” regarding risk of bias, cohort studies ranged from medium to low quality, and most pre-clinical studies had high bias risk. sCAS significantly reduced root damage compared to freehand methods (OR = 0.11; 95% CI: 0.04–0.36; *p* < 0.001; I² = 1%) and reduced angular and linear deviations. Due to heterogeneity, no quantitative synthesis of accuracy outcomes was performed. **Conclusions**: sCAS improves the safety and accuracy of MS insertion compared to freehand and radiographic guide methods. These results highlight the clinical benefits of sCAS in orthodontics. Future studies should refine protocols and explore dCAS for further accuracy improvements.

## 1. Introduction

Obtaining enough anchorage is a primary objective in orthodontics, as it facilitates the desired tooth movement and prevents unwanted movements [1]. Traditionally, anchorage has been provided through intraoral and extraoral points, such as teeth, the palatal vault, extraoral arches, or facemasks [2].

Nowadays, Temporary Anchorage Devices (TADs), including miniscrews (MSs) and miniplates, have simplified many conventional orthodontic procedures and minimized the undesirable effects of several appliances by providing skeletal anchorage [3]. TADs can be placed either in the alveolar ridge as interradicular MSs or away from the roots, such as in the infrazygomatic crest, the lower nasal spine, or the mandibular ramus [1].

Interradicular MSs carry the risk of complications, such as periodontal ligament or root surface injury and lack of primary stability [4]. Traditionally, MSs have been placed using a freehand approach, relying on clinical and anatomical landmarks to guide the insertion point and axis [5,6,7]. However, this technique often results in significant deviations, and the lack of space increases the risk of iatrogenic root damage [4]. To overcome such drawbacks, alternative placement methods, such as using radiopaque markers or manufacturing resin guides from plaster models, have been developed. Nevertheless, these “oriented” approaches to insert MSs seem to offer limited accuracy [8,9,10].

Recent improvements in three-dimensional (3D) imaging techniques, including Cone Beam Computed Tomography (CBCT), have addressed the limitations of two-dimensional imaging techniques, allowing for accurate assessment of the interradicular space and available bone. With the advent of 3D planning software, static Computer-Assisted Surgery (sCAS) using stereolithography 3D-printed guides can enhance the precision of MS insertion. While most literature on CAS focuses on dental implants, studies indicate that it can also be effective in ensuring precise insertion of miniscrews (MS) at the correct angle and depth, significantly reducing the risk of iatrogenic injuries [11,12]. Another approach reported in implantology is dynamic Computer-Assisted Surgery (dCAS), which employs tracking technology to monitor and provide real-time feedback on the intraoperative position of the surgical instruments and the patient. These navigation systems offer valuable guidance for safer oral and maxillofacial surgery, demonstrating greater accuracy compared to conventional or freehand techniques [13,14,15,16].

The scientific evidence on computer-assisted orthodontic MS placement remains limited. A recent systematic review and network meta-analysis by Mihit et al. [17] sheds some light; however, it exclusively includes in vitro studies and focuses on MS placements in the anterior palate. To address this gap, the present systematic review aims to comprehensively evaluate the safety and accuracy of interradicular orthodontic MS placement using different guidance approaches.

## 2. Materials and Methods

This study adhered to the Preferred Reporting Items for Systematic Reviews and Meta-analysis (PRISMA) statement [18] and was registered in the international database of prospectively registered systematic reviews in health and social care (PROSPERO) under number CRD42022321297.

The focused question to be addressed was as follows: For patients undergoing orthodontic treatment or using pre-clinical models (i.e., resin models, cadavers, or animals) that require the placement of at least one interradicular MS, what are the safety and accuracy of MS placement using any instrument or stent to guide their insertion?

### 2.1. Search Strategy

An electronic search was conducted in PubMed (MEDLINE), Scopus (Elsevier), The Cochrane Library (Wiley), and Web of Science (Clarivate Analytics) up to 4 March 2024, to identify all relevant studies without applying data or language restrictions (Supplementary Table S1). Additionally, grey literature was searched on OpenGrey (OpenGrey 2011), as well as the US National Institutes of Health (National Institutes of Health, 2000), in order to identify additional potential candidates to be included. The research was further supplemented by reviewing the reference lists of the selected articles and reviews [17].

### 2.2. Selection and Data Collection Processes

All published studies evaluating the safety and/or accuracy of orthodontic MSs placed in the buccal interradicular area using any type of guidance were included, regardless of the MS type. Eligible studies comprised randomized (RCT) or non-randomized clinical trials, case-control studies, prospective or retrospective cohort studies, case series, animal, ex vivo, and in vitro studies with at least 10 interradicular MSs. Case reports or technical notes were excluded. Additionally, studies assessing MS accuracy or safety without any guidance or those examining MSs placed in locations other than the interradicular space (e.g., palatal vault, retromolar region, infrazygomatic crest, or the nasal process) were also excluded.

Two independent researchers (MSO and AJG) screened the studies in accordance with the eligibility criteria. Any disagreements were resolved by discussion with a third reviewer (OCF). The Cohen kappa coefficient (κ) was calculated to measure the level of agreement of the reviewers.

Initially, duplicates or irrelevant publications (based on the title) were excluded, followed by an examination of the abstracts. Finally, the full texts of all the remaining papers were assessed. The studies removed at this stage and the reasons for their exclusion were recorded.

Authors were contacted when necessary to clarify missing information. In cases where multiple reports on the same patients were identified, the most recent report was included.

### 2.3. Data Items

The primary outcome, safety, was assessed by the risk of root contact or damage during the surgical procedure. The secondary outcome, accuracy, was evaluated by comparing the preoperative virtual surgical plan with the final MS position, focusing on angular, coronal, and apical deviations (see Table 1 for definitions).

### 2.4. Study Risk of Bias Assessment

Two reviewers (MSO and TMM) independently assessed the risk of bias and quality of the included studies.

RCTs were evaluated using the Revised Cochrane risk-of-bias tool for randomized trials (RoB 2) [updated 22 August 2019] [19], which assesses five domains: the randomization process, deviations from intended interventions, missing outcome data, measurement of the outcome, and selection of the reported result. Each domain was rated as “low” risk, “high” risk, or “some concerns”.

Prospective or retrospective cohort studies were assessed using the Newcastle–Ottawa Scale (NOS) [20], which evaluates three domains: Selection, Comparability, and Outcome. The studies could receive up to 9 stars, with 8–9 stars indicating high methodological quality, 6–7 stars indicating medium-level quality, and 5 stars or fewer indicating low quality. For studies with only one exposed group (without comparison), one question from the Selection domain and the entire Comparability domain were omitted, allowing for a maximum of 6 stars.

The risk of bias in pre-clinical studies was evaluated using an adapted tool from previous research [21,22,23], which assessed 10 parameters, including the presence of a control group, sample size calculation, randomization, and blinding. Articles were then classified as having a high risk of bias (1–4 items reported), medium risk (5–7 items reported), or low risk (8–10 items reported).

### 2.5. Assessment of the Certainty of the Evidence

The certainty of the evidence was assessed using the GRADE (Grading of Recommendations Assessment, Development, and Evaluation) framework. This evaluation considered several key factors, including the overall risk of bias in the included studies, the consistency of the results, the directness of the evidence, the precision of the effect estimates, and the potential for publication bias.

### 2.6. Data Extraction and Method of Analysis

Two reviewers (MSO and AJG) independently extracted the qualitative data using data-extraction tables. The following information in each study was retrieved: authors, year and country of publication, study design, number of subjects (patients, cadavers, phantoms, or animals) and MSs, MS type and dimensions, groups assessed, preoperative planning method, post-operative evaluation method, studied variables, number of root contacts and failures, and quantitative deviations.

The MS placement methods were divided into three categories:

- dCAS group: Surgical navigation technology with real-time tracking of the intraoperative position of the surgical instrument and the patient on CBCT images.
- sCAS group: Surgical templates fabricated using stereolithographic computer-aided design and manufacturing (CAD-CAM) techniques from virtual simulations of the MS placement on 3D models reconstructed from CBCT or CT images.
- Radiographic guide group: Wire guides, grids, or any other partially guided devices used as radiopaque references in preoperative evaluation, regardless of whether the guide was used during the surgical procedure.

If a study compared the intervention group with a control group using a freehand approach, data from the freehand group were also retrieved to compare the results of the studies.

### 2.7. Statistical Analysis

The statistical analysis was performed using Review Manager software (Review Manager version 5.3; The Cochrane Collaboration, Copenhagen, Denmark), with the MS serving as the statistical unit.

A meta-analysis using random-effects models was conducted only when there were studies reporting on common parameters. Accordingly, quantitative synthesis of the accuracy outcomes was not conducted due to substantial heterogeneity among the included studies.

For the safety analysis, the pooled event rate and its 95% confidence interval (95% CI) were calculated using logit-transformed proportions. Additionally, pairwise meta-analyses were performed for studies that directly compared different MS placement methods. Odds ratios (ORs) with 95% CI were used to estimate the effect of the intervention. Statistical significance was defined as *p* < 0.05 for all analyses.

Statistical heterogeneity was estimated by means of χ^2^ (Q value) and I^2^ analyses. A χ^2^ *p*-value of <0.10 and an I^2^ value of >50% were interpreted as significant heterogeneity [24].

Had there been a sufficient number of meta-analyzed studies (more than 10), publication bias, clinical heterogeneity assessment, and sensitivity analyses would have been performed according to Patsopoulos et al. [25].

## 3. Results

### 3.1. Study Selection

**Figure 1:**
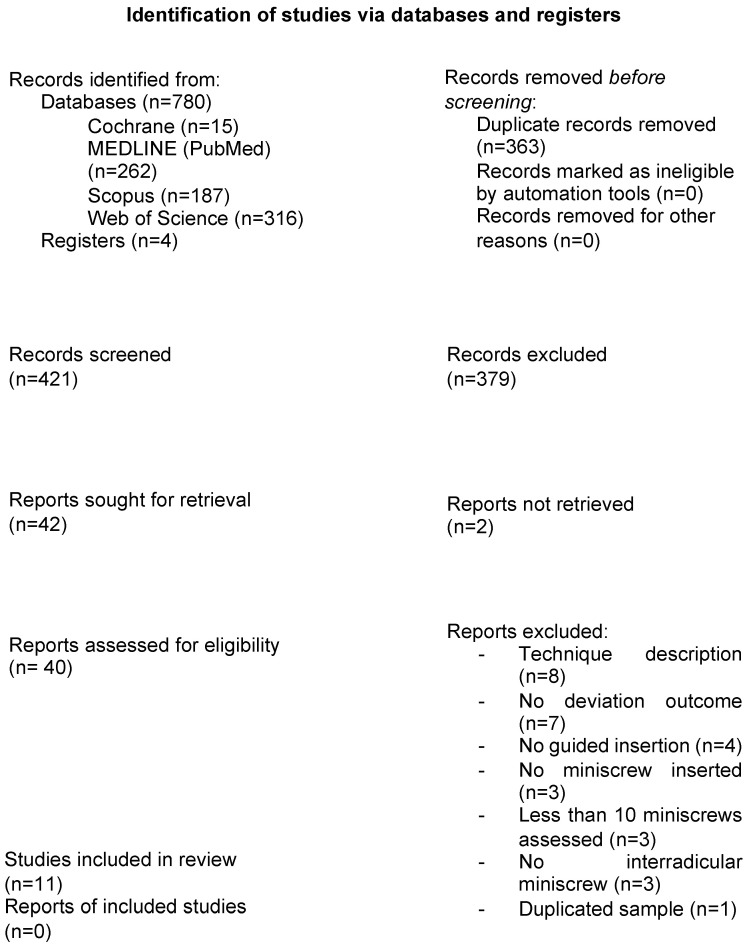
PRISMA 2020 flow diagram of the screening and selection process.

The initial electronic database search yielded 763 references, with 4 additional articles identified through manual searching. Thirty-one articles were excluded after full-text evaluation (Table 2). Altogether, 11 studies [12,26,27,28,29,30,31,32,33,34,35] were selected for qualitative and quantitative synthesis. The reviewer agreement rate was 95%, with a κ coefficient of 0.87 (almost perfect agreement). A flowchart of the screening process is shown in Figure 1.

### 3.2. Study Characteristics

The 11 included studies [12,26,27,28,29,30,31,32,33,34,35] provided a total of 652 MSs. These comprised two in vitro studies [32,33], one animal study [31], one cadaver study [30], and seven human studies [12,26,27,28,29,34,35]. Among the human studies, there was one split-mouth RCT [35], five prospective cohort studies [12,27,28,29,34], and one retrospective cohort study [26].

The distribution of MS placements by method was as follows: 182 placements using sCAS [12,28,29,30,32,33], 283 placements with radiographic guides [26,27,31,34,35], and 139 placements using the freehand technique [26,30,32,33,35]. No studies evaluating dCAS were identified. Detailed results for each individual study are presented in Table 2.

### 3.3. Risk of Bias and Quality Assessment

The only RCT included in the review [35] was assessed as having “some concerns” regarding overall risk of bias, particularly due to issues related to randomization, deviations from the intended intervention, and selective reporting of results. Cohort studies comparing exposed and non-exposed groups were rated as medium quality [26] and low quality [34]. In contrast, cohort studies that only examined a single exposed group [12,27,28,29] were consistently judged to be of low quality. All pre-clinical studies exhibited a high risk of bias [30,31,32], with the exception of one study [33], which was assessed as having a medium risk of bias.

**Figure 2:**
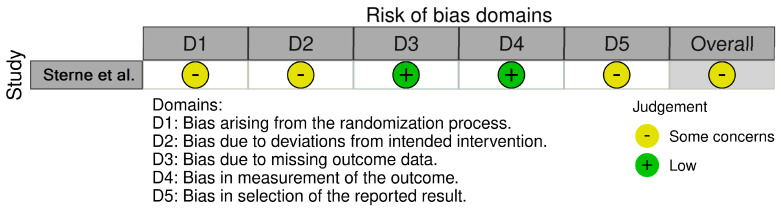
Risk of bias assessment of the RCT included (Cochrane Collaboration tool) [19].

Figure 2 and Table 3 and Table 4 summarize the quality assessments of the included articles.

### 3.4. Qualitative Synthesis

The safety analysis included nine studies [12,26,28,29,30,32,33,34,35] involving 542 interradicular MSs. A total of 33 root contacts were recorded: 4 in the sCAS group (182 MSs; weighted risk = 0.56%; 95%CI: 0 to 4.87), 3 in the radiographic guide group (241 MSs; weighted risk = 1.55%; 95%CI: 0 to 13.61), and 26 in the freehand group (119 MSs; weighted risk = 21.38%; 95%CI: 3.13 to 47.84).

Angular and linear deviations were defined variably across the studies, with measurements taken at the head and tip of the MS and in different directions (i.e., buccopalatal, mesiodistal, and/or vertical), using both 2D (different RX projections) and 3D imaging systems. Due to the significant heterogeneity in the methods used to evaluate accuracy, statistical analysis was not feasible, and direct comparisons between the studies could not be made. However, qualitative data for the sCAS and radiographic guide groups are provided in Table 5 and Table 6, respectively. Overall, both the sCAS [30,32,33] and radiographic guide [34] groups were associated with lower apical, coronal, and angular deviations compared to the freehand placement technique.

In the sCAS group, all six studies employed a fully guided insertion sequence using tooth-supported stereolithography 3D-printed guides. Among the sCAS group studies that reported linear deviations [28,29,30,33], these ranged from 0.73 mm to 1.06 mm and 0.73 mm to 1.26 mm in the apical and coronal, respectively. Angular deviations ranged from 1.76° to 4.66°, with the lowest angular deviation reported by Morea et al. [28] at 1.76° (SD = 0.93).

In the radiographic guide group, all studies employed different types of custom-made guides (e.g., grid, pin, and wire). Coronal deviations ranged from 0.17 mm (SD = 0.11) [27] to 1.00 mm (SD = 0.40) [26], while apical inaccuracies varied from 1.04 mm [35] to 5.30 mm [26]. Angular deviations also displayed a broad range, from as low as 1.80° (SD = 0.90) to as high as 16.90° (SD = 2.60) [26].

### 3.5. Assessment of the Certainty of the Evidence

In accordance with the GRADE framework, the certainty of the evidence for the primary outcome was assessed as very low. A detailed Summary of Findings (SoF) table, providing a comprehensive overview of the evaluation, is available as Supplementary Materials (Supplementary Table S2).

### 3.6. Quantitative Synthesis

**Figure 3:**
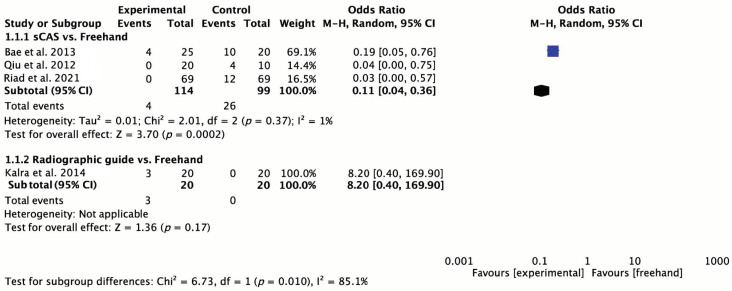
Forest plot for safety (primary outcome) [30,32,33,35].

The sCAS group was associated with a decreased rate of iatrogenic root damage compared to the freehand technique (OR = 0.11; 95%CI: 0.04 to 0.36; *p* < 0.001; I^2^ = 1%). Conversely, the use of radiographic guides did not prove better than the freehand approach (OR = 8.20; 95%CI: 0.40 to 169.90; *p* < 0.001; I^2^ = Not applicable) (Figure 3).

## 4. Discussion

This review aimed to comprehensively summarize the available data on oriented and guided interradicular MS insertion, with a focus on accuracy and safety. Indeed, several methods have been described in the literature to improve the accuracy and safety of MS placement, which are critical factors in minimizing failure rates [4,36]. Our findings indicate that sCAS methods significantly enhance the accuracy of MS placement, aligning the final screw position more closely with the preoperative plan while reducing the risk of root damage compared to radiographic guides and freehand techniques.

Accurate insertion of miniscrews (MS) is crucial in orthodontic treatment to avoid root contact and prevent iatrogenic dental lesions, given the limited interradicular space. Root contact can not only endanger the affected tooth but also compromise the stability of the MS, potentially undermining the success of the treatment [4]. In this sense, Alharbi et al. [37] reported a failure rate of 13.5% (95%CI: 11.5 to 15.9). Additional factors influencing MS failure include MS angle and insertion point, soft tissue conditions, cortical bone thickness and quality, MS characteristics, primary stability, load intensity, oral hygiene, and surrounding tissue inflammation [36,38,39,40,41].

Traditionally, clinical parameters have guided freehand MS insertion. In the upper jaw, MS placement 4 to 6 mm below the alveolar crest and with a vertical insertion angle of 30° to 45° relative to the tooth axis has been recommended to avoid roots and ensure sufficient cortical bone for stability [40,42]. However, several studies with freehand groups did not specify the clinical parameters used [26,30,32,33,35]. In any case, according to the included studies, root damage occurred in more than 20% of cases using the freehand technique (weighted risk = 21.38%; 95%CI: 3.13 to 47.84) [26,30,32,33,35]. Additionally, greater angular and linear deviations were observed compared to guided approaches, indicating that clinical landmarks alone may not be reliable. Contributing factors to these results include anatomical variability among patients, differences in clinician skill and experience, and limited visibility during the procedure [30].

Radiographic guides and non-fully guided or “oriented” stents have been proposed to improve accuracy. Although studies using these approaches showed better accuracy than freehand techniques, the deviations between the planned and final positions were still notable, with figures reaching 5.3 mm (SD = 1.1) in apical deviation and 16.9° (SD = 2.6) in angular deviation [26]. Moreover, our meta-analysis revealed that radiographic guides did not significantly reduce root damage in comparison with freehand techniques (OR = 8.20; 95%CI: 0.40 to 169.90; *p* < 0.001). Although radiographic guides are affordable and easy to use, they provide only limited two-dimensional information at the insertion point. This limitation can lead to inconsistent relative positions across different radiographic projections, contributing to the inaccuracies observed in the MS placement [8,9,10]. Accordingly, radiographic guides may not be sufficiently reliable for precise interradicular MS placement when the space is limited.

sCAS has been widely used in dental implant placement to enhance accuracy. A previous meta-analysis [43] assessing sCAS for dental implants reported a mean angular deviation of 3.3° (95% CI: 2.07 to 4.63) and mean deviations at the entry point and apical positions of 0.9 mm (95% CI: 0.79 to 1.00) and 1.2 mm (95% CI: 1.11 to 1.20), respectively. As expected, similar accuracy outcomes were observed in the present review, because both dental implants and orthodontic MSs rely on self-tapping screws. Specifically, the sCAS method demonstrated coronal, apical, and angular deviations as low as 0.73 mm (range: 0.26 to 1.12 mm), 0.73 mm (range: 0.24 to 2.07 mm), and 1.76° (SD = 0.93), respectively [28,30]. In terms of safety, the sCAS group exhibited significantly less iatrogenic root damage in comparison with the freehand technique (OR = 0.11; 95% CI: 0.04 to 0.36; *p* < 0.001; I² = 1%). Evidence from both clinical and pre-clinical studies consistently shows that sCAS significantly improves safety in MS insertion [12,28,30,32], particularly when performed by novice clinicians. sCAS might reduce operator variability, enabling less experienced practitioners to achieve consistent results, and improve accuracy, reducing the gap between unexperienced clinicians and experts. However, despite its proven precision, accuracy, and safety, sCAS does have certain limitations. These include the complexity of the fabrication process, longer preparation times, higher costs, and increased radiation exposure compared to 2D radiographs [35,44]. Additionally, patients with existing prosthetic restorations, such as crowns or temporary veneers, may present challenges during CBCT imaging due to the presence of radiographic artifacts. These artifacts can obscure anatomical details and compromise the accuracy of the preoperative plan, potentially contributing to deviations in MS placement and reducing overall precision. Furthermore, sCAS lacks intraoperative flexibility, requires specific tools, limits access, and reduces tactile feedback during the procedure [45].

To overcome some of the aforementioned limitations, dCAS technology has been proposed. To date, three studies have evaluated dCAS for orthodontic MS insertion, with accuracy results comparable to those reported for dental implants [45,46,47]. However, no studies have specifically examined its use for interradicular MS placement, highlighting the need for further research to validate these findings in this context.

This review faced several challenges, including the inclusion of pre-clinical studies [29,30,31,32] and only one RCT [35]. Many studies presented a potential risk of bias [12,27,28,29,30,31,32,34], and there was significant heterogeneity in deviations and accuracy, which precluded quantitative synthesis of the data. Consequently, more research is required to standardize methods for measuring deviations and to evaluate the long-term benefits and cost-effectiveness of CAS in MS insertion. Additionally, future studies should explore advanced technologies, such as robotic or dCAS technologies, to further enhance accuracy, precision, and safety.

## 5. Conclusions

The insertion method for interradicular orthodontic MSs significantly impacts their precision, accuracy, and safety. Evidence suggests that sCAS techniques offer superior accuracy and precision, along with a reduced risk of root damage, compared to radiographic guides or freehand placement of interradicular MSs.

## Figures and Tables

**Table 1:** Overview of data items.

	Variable		Definition
**Primary outcome**	Safety		Risk of root contact or damage during the surgical procedure
**Secondary outcome**	Accuracy	Angular deviation	Difference in the angulation of the MS compared to the planned insertion angle
		Coronal deviation	Difference in the MS position from the planned position at the coronal level
		Apical deviation	Difference in the MS position from the planned position at the apical level

MS: miniscrew.

**Table 2:** Summary of the characteristics of the included studies.

Authors (Year)	Country	Design	No. of Subjects MSs	MS Type and Dimensions	Groups	Pre-Operative Planning Method	Post-Operative Planning Method
Bae et al. (2013) [30]	South Korea	Cadaver study	12–45	BMK; Biomaterials Korea, Seoul, Republic of Korea (1.5 × 7 mm)	- sCAS (25 MS) - FH (20 MS)	- CAS: CBCT + 3D planning software - FH: intraoral radiograph.	Pre and post CBCT overlapping + 3D analysis program
Estelita et al. (2009) [27]	Brazil	Cohort prospective study	6–10	SH 1514-07, Absoanchor, Dentos, Daegu, Republic of Korea (1.5 × 7 mm)	Radiographic guide	Intraoral radiograph	Intraoral radiograph
Kalra et al. (2014) [35]	India	RCT split-mouth	13–40	DB Orthodontics Limited, West Yorkshire, UK (1.5 × 9 mm)	- FH (20 MS) - Radiographic guide (20 MS)	- FH: CBCT - Radiographic guide: intraoral radiograph	CBCT without overlapping
Liu et al. (2010) [12]	China	Cohort prospective study	11–34	Beici Medical Company, Ningbo, China (1.6 × 11mm)	sCAS	CBCT + 3D planning software	Pre and post CBCT overlapping + 3D analysis program
Morea et al. (2011) [28]	Brazil	Cohort prospective study	4–10	Dentaurum, Ispringen, Germany (NR × 6–8 mm)	sCAS	CBCT + 3D planning software	Pre and post CBCT overlapping + 3D analysis program
Qiu et al. (2012) [32]	China	In vitro study	3–30	Ortholution, Seoul, Republic of Korea (1.8 × 7 mm)	- sCAS (20 MS) - FH (10 MS)	CBCT + 3D planning software	Pre and post CBCT overlapping + 3D analysis program
Qiu et al. (2021) [29]	China	Cohort prospective study	10–24	Ningbo Cibei Medical Equipment Co. LTD., China (1.6 × 9 mm)	sCAS	CBCT + 3D planning software	Pre and post CBCT overlapping + 3D analysis program
Riad et al. (2021) [33]	Spain	In vitro study	14–207	Dual Top^®^ Anchor System, JEIL Medical Corporation, Guro-gu, Seoul, Republic of Korea (1.3 × 8 mm)	- sCAS (69 MS) - Mixed reality device (69 MS) - FH (69 MS)	CBCT + 3D planning software	Pre and post CBCT overlapping + 3D analysis program
Suzuki et al. (2008) [26]	Thailand	Cohort retrospective study	NR–220	NR	- Radiographic guide 1 (custom-made sleeve guide) (180 MS) - Radiographic guide 2 (wire guide) (20 MS) - FH (20 MS)	Intraoral radiograph	Intraoral radiograph
Thakur et al. (2012) [34]	India	Cohort prospective study	21–21	NR (1.3 × 8 mm)	- Radiographic guide 1 (grid) (7 MS) - Radiographic guide 2 (X-ray pin) (7 MS) - Radiographic guide 3 (wire eye) (7 MS)	Intraoral radiograph	Intraoral radiograph
Yu et al. (2012) [31]	South Korea	Animal study	4–32	C-implant, C-implant Company, Seoul, Republic of Korea (1.8 × 8.5 mm)	Radiographic guide	CBCT and manually transferring the planning to the surgical stent	CBCT without overlapping

FH, freehand; CBCT, Cone Beam Computed Tomography; MS, miniscrew; NR, not reported; RCT, Randomized Clinical Trial; sCAS, static Computer-Assisted Surgery.

**Table 3:** Risk of bias assessment of cohort studies (NOS).

Study	Selection	Comparability	Outcome	NOS Score and Overall Quality Assessment
Estelita et al. [27]	**	N/A	**	4 (low quality)
Liu et al. [12]	***	N/A	**	5 (low quality)
Morea et al. [28]	***	N/A	**	5 (low quality)
Suzuki et al. [26]	****	-	**	6 (medium quality)
Thakur et al. [34]	**	-	**	4 (low quality)
Qiu et al. [29]	***	N/A	**	5 (low quality)

N/A: not applicable; (-): no points awarded; (**): 2 points; (***): 3 points; (****): 4 points.

**Table 4:** Risk of bias assessment of pre-clinical studies.

Study	Control	Sample Size Calculation	Randomization	Allocation Concealment	Same Experimental Conditions	Properly Described Intervention
Bae et al. [30]	Y	N	N	N	N	Y
Qiu et al. [32]	Y	N	N	N	Y	Y
Riad et al. [33]	Y	N	Y	N	Y	Y
Yu et al. [31]	N	N	N/A	N/A	N/A	Y
**Study**	**Same Operator**		**Blinding Operator**	**Blinding Outcome Assessor**	**Incomplete** **Outcome**	**Overall** **Assessment**
Bae et al. [30]	Unclear		N	Unclear	Unclear	High risk
Qiu et al. [32]	Unclear		N	Unclear	Unclear	High risk
Riad et al. [33]	Y		N	Unclear	Unclear	Medium risk
Yu et al. [31]	N		N	Unclear	Unclear	High risk

Y: yes; N: no; N/A: not applicable.

**Table 5:** Deviation information for studies reporting sCAS.

Authors (Year)	Studied Variables	No. of Root Contacts	Deviations
Bae et al. [30]	3D angular deviations (°) and 3D distance (coronal and apical) deviations (mm); root contact or damage	sCAS: 4/25 (contact) FH: 6/20 (contact) and 4/20 (damage)	3D coronal deviations *: - sCAS: 0.73 mm (range: 0.26 to 1.12 mm) - FH: 1.56 mm (range: 0.59 to 2.95 mm) 3D apical deviations *: - sCAS: 0.73 mm (range: 0.24 to 2.07 mm) - FH: 1.28 mm (range: 0.26 to 3.81 mm) 3D angular deviations: - sCAS: 3.14° (range: 1.02 to 10.9°) - FH: 9.57° (range: 3.15 to 35.6°)
Liu et al. [12]	Angular (°) and lineal (mm) deviations measured at the apex and placement sites (reported in 3 directions: distomesial, vertical, and buccopalatal)	NR– All deviations of the miniscrews were in the safe zone (less than 0.86 ± 0.125 mm)	Coronal deviation: - 0.1 ± 0.01 mm Apical deviations: - Mesiodistal direction: 0.42 ± 0.13 mm * - Vertical direction: 0.47 ± 0.12 mm - Buccopalatal directions: 0.59 ± 0.26 mm - Angular deviations: - Mesiodistal direction: 1.2 ± 0.43° - Vertical direction: 1.3 ± 0.41° - Buccopalatal directions: 1.6 ± 0.79°
Morea et al. [28]	3D angular (°) deviation and distance (coronal, central, and apical) deviations, root contact	0/10	3D coronal deviation: 0.86 ± 0.57 mm 3D central deviation: 0.71 ± 0.34 mm 3D apical deviation: 0.87 ± 0.54 mm Angular deviation: 1.76 ± 0.93°
Qiu et al. [32]	Angular (°) and lineal (mm) deviations measured at the apex and placement sites (reported in two directions: mesiodistal and vertical), root contact	FH: 4/10 sCAS: 0/20	Coronal deviations: - Vertical direction *: ○ sCAS: 0.19 ± 0.19 mm ○ Freehand: 0.94 ± 0.87 mm - Mesiodistal direction *: ○ sCAS: 0.15 ± 0.09 mm ○ Freehand: 0.48 ± 0.46 mm Apical deviations: - Vertical direction *: ○ sCAS: 0.33 ± 0.25 mm ○ FH: 0.78 ± 0.49 mm - Mesiodistal direction *: ○ sCAS: 0.28 ± 0.23 mm ○ FH: 0.81 ± 0.61 mm Angular deviations: - Mesiodistal direction *: ○ sCAS: 1.47 ± 0.56 ° ○ FH: 7.49 ± 6.09 ° - Vertical direction *: ○ sCAS: 2.13 ± 1.48° ○ FH: 6.31 ± 3.82°
Qiu et al. [29]	3D distance (coronal and apical) deviations (mm); root contact	0/24	- 3D coronal deviation: 1.03 ± 0.65 mm - 3D apical deviation: 1.26 ± 0.72 mm
Riad et al. [33]	3D angular (°) deviation and distance (coronal and apical) deviations (mm); root contacts	sCAS: 0/69 Mixed-reality: 0/69 FH: 12/69	3D coronal deviation *: - sCAS: 1.06 ± 0.59 mm - Mixed-reality: 1.74 ± 0.52 mm - FH: 2.2 ± 2.0 mm * Statistically significant differences (*p* < 0.001) between sCAS and mixed-reality, sCAS and freehand, and mixed-reality and freehand. 3D apical deviation *: - sCAS: 1.11 ± 0.77 mm - Mixed-reality: 1.86 ± 0.65 mm - FH: 1.69 ± 0.82 mm * Statistically significant differences (*p* < 0.001) between sCAS and mixed-reality and sCAS and FH: 3D angular deviation *: - sCAS: 4.66 ± 3.65 mm - Mixed-reality: 5.5 5± 2.46 mm - FH: 7.58 ± 3.5 mm * Statistically significant differences (*p* < 0.001) between sCAS and mixed-reality and sCAS and FH.

* Statistically significant differences (*p* < 0.05). FH: freehand; sCAS, static Computer Assisted Surgery; NR, not reported.

**Table 6:** Deviation information for studies reporting radiographic guide.

Authors (Year)	Studied Variables	No. of Roots Contacts	Deviations
Estelita et al. [27]	Miniscrew centralization degree, inaccuracy degree, and risk index	NR	Mesiodistal lineal deviation (inaccuracy degree): 0.17 ± 0.11 mm
Kalra et al. [35]	Height deviation (mm), angular mesiodistal (°), and distance mesiodistal deviations (mm) measured at the coronal and apical areas; root contact	Radiographic guide: 3/20 FH: 0/20	Height deviation: - Radiographic guide: 0.56 mm * - FH: 0.1 mm * Coronal deviation (mesiodistal direction): - Radiographic guide: 0.65 mm - FH: 0.39 mm Apical deviation (mesiodistal direction): - Radiographic guide: 1.04 mm - FH: 0.59 mm Angular deviation (mesiodistal direction): - Radiographic guide: 6.2° - FH: 4.7°
Suzuki et al. [26]	Angular deviation (°) and distance deviation (mm) measured at the head and tip	Radiographic guide 1 (custom-made sleeve guide): 0/180 (damage) Radiographic guide 2 (wire guide): 0/20 (damage) FH: NR	2D Coronal deviation *: - Radiographic guide 1: 0.6 ± 0.4 mm - Radiographic guide 2: 1.0 ± 0.4 mm - FH: 3.6 ± 1.4 mm 2D Apical deviation *: - Radiographic guide 1: 2.0 ± 0.4 mm - Radiographic guide 2: 5.3 ± 1.1 mm - FH: 10.5 ± 3.5 mm 2D Angular deviations *: - Radiographic guide 1: 1.8 ± 0.9° - Radiographic guide 2: 16.9 ± 2.6° - FH: 21.2 ± 2.9° * Statistically significant differences (*p* < 0.001)
Thakur et al. [34]	Horizontal distances: PDL-PDL, PIP-GC, PIP-nearest root, AIP-GC, AIP-nearest root; vertical distance: PIP-AC, AIP-AC; number of radiographs required Pre, post, and differences between pre-post	Radiographic guide 1 (grid): 0/7 Radiographic guide 2 (X-ray pin): 0/7 Radiographic guide 3 (wire eye): 0/7 NR– All deviations of the miniscrews were in the safe zone	Horizontal distance PIP-GC (preoperative) * - Grid guide: 0.15 ± 0.16 - X-ray pin guide: 0.28 ± 0.10 - Wire eye guide: 0.38 ± 0.07 Vertical level PIP-AC (preoperative) - Grid guide: 5.23 ± 0.52 - X-ray pin guide: 5.02 ± 0.56 - Wire eye guide: 5.12 ± 0.46 Horizontal level between pre- and postoperative - Grid guide: 0.18 ± 0.18 - X-ray pin guide: 0.23 ± 0.26 - Wire eye guide: 0.19 ± 0.12 Vertical level between pre- and postoperative - Grid guide: 0.17 ± 0.06 - X-ray pin guide: 0.25 ± 0.11 - Wire eye guide: 0.26 ± 0.10 Horizontal distance AIP-nearest root - Grid guide: 0.22 ± 0.18 - X-ray pin guide: 0.18 ± 0.26 - Wire eye guide: 0.19 ± 0.12
Yu et al. [31]	Vertical and horizontal angulation between prescribed and final mini-implant positions	NR	- Horizontal angular deviation (transversal view): 1.16 ± 6.08° - Vertical angular deviation (coronal view): 1.01 ± 7.25°

AC: alveolar crest; AIP: actual insertion point; FH: freehand; GC: center of neighboring roots; NR: not reported; PDL: periodontal ligament; PIP: planned insertion point. * Statistically significant differences (*p* < 0.05)

## Data Availability

Data are contained within the article.

## Supplementary Materials

The following supporting information can be downloaded at https://www.mdpi.com/article/10.3390/jcm13247697/s1. Table S1. Database search strategy. Table S2. Summary of Findings (GRADE). Table S3. Excluded studies and reasons for exclusion.
