# Supplementary material for: Safety and Accuracy of Guided Interradicular Miniscrew Insertion: A Systematic Review and Meta-Analysis
Source: J Clin Med. 2024 Dec 17;13(24):7697. doi: 10.3390/jcm13247697 (PMC11678679; doi:10.3390/jcm13247697)
Supplement: Supplementary file 1 [file jcm-13-07697-s001.zip › jcm-3313545-supplementary.pdf]

Supplementary Materials:

Table S1. Database search strategy.

Database	Search (March 4th, 2024)
Cochrane Library (Wiley)	**1 Title Abstract Keyword** MeSH descriptor: [Orthodontic Anchorage Procedures]<br>**2 Title Abstract Keyword** "Miniscrew" or "Mini screw" or mini-screw* or miniimplant* or "mini implant" or "Temporary anchorage" or TADs or TAD or "skeletal anchorage" or bone anchorage<br>**3 Title Abstract Keyword** Orthodon*<br>**4 Title Abstract Keyword** (MeSH descriptor: [Surgery, Computer-Assisted]) OR ("computer navigation system" OR "computer guided surgery" OR "surgical guide" OR "surgical stent" OR "surgical template" OR "accuracy" OR "free-hand" OR "freehand" OR "conventional technique" OR "conventional procedure" OR "conventional surgical guide" OR "conventional free-hand method" OR "radiographic reference" OR "periapical radiograph" OR "panoramic radiograph" OR "radiographic surgical guide" OR "radiographic guide" OR "cast" OR "wire guide" OR "visible guide")<br>**(#1 OR #2) AND #3 AND #4**
MEDLINE (PubMed)	("Orthodontic Anchorage Procedures"[Mesh] OR (Miniscrew* OR "Mini screw*" OR Mini-screw* OR Mini- implant* OR "Mini implant*" OR "Temporary anchorage*" OR TAD OR TADs OR "skeletal anchorage" OR "bone anchorage")) AND Orthodon* AND ("Surgery, Computer-Assisted" [Mesh] OR "computer navigation system" OR "computer guided surgery" OR "surgical guide" OR "surgical template" OR "Dimensional Measurement Accuracy"[Mesh] OR "accuracy" OR "free-hand" OR "freehand" OR "conventional technique*" OR "conventional procedure*" OR "conventional surgical guide*" OR "conventional free-hand method*" OR "radiographic reference*" OR "periapical radiograph*" OR "panoramic radiograph" OR "radiographic surgical guide" OR "radiographic guide" OR "cast*" OR "wire guide" OR "visible guide")
Scopus (Elsevier)	TITLE-ABS-KEY ("Orthodontic Anchorage Procedures" OR Miniscrew* OR "Mini screw*" OR Mini-screw* OR Mini- implant* OR "Mini implant*" OR "Temporary anchorage*" OR TAD OR TADs OR "skeletal anchorage" OR "bone anchorage") AND TITLE-ABS-KEY (Orthodon*) AND TITLE-ABS-KEY ("Surgery, Computer-Assisted" OR "computer navigation system" OR "computer guided surgery" OR "surgical guide" OR "surgical stent" OR "surgical template" OR “dimensional measurement accuracy” OR "accuracy" OR "free-hand" OR "freehand" OR "conventional technique*" OR "conventional procedure*" OR "conventional surgical guide*" OR "conventional free-hand method*" OR "radiographic reference*" OR "periapical radiograph*" OR "panoramic radiograph" OR "radiographic surgical guide" OR "radiographic guide" OR "cast*" OR "wire guide" OR "visible guide")
Web of Science (Clarivate)	((TS=("Orthodontic Anchorage Procedures" OR "Miniscrew*" OR "Mini screw*" OR "Mini-screw*" OR "Mini- implant*" OR "Mini implant*" OR "Temporary anchorage*" OR "TAD" OR "TADs" OR "skeletal anchorage" OR "bone anchorage")) AND TS=(Orthodon*)) AND TS=("Surgery, Computer-Assisted" OR "computer navigation system" OR "computer guided surgery" OR "surgical guide" OR "surgical stent" OR "surgical template" OR “dimensional measurement accuracy” OR "accuracy" OR "free-hand" OR "freehand" OR "conventional technique*" OR "conventional procedure*" OR "conventional surgical guide*" OR "conventional free-hand method*" OR "radiographic reference*" OR "periapical radiograph*" OR "panoramic radiograph" OR "radiographic surgical guide" OR "radiographic guide" OR "cast*" OR "wire guide" OR "visible guide")

Table S2. Summary of Findings (GRADE).

Certainty assessment							№ of patients		Effect		Certainty	Importance
**№ of studies**	**Study design**	**Risk of bias**	**Inconsistency**	**Indirectness**	**Imprecision**	**Other considerations**	**sCAS**	**Freehand**	**Relative (95% CI)**	**Absolute (95% CI)**		
**Safety (root contact or damage) - sCAS vs Freehand**												
3	non-randomised studies	very serious	not serious	very serious[a]	extremely serious[b]	none	4/114 (3.5%)	26/99 (26.3%)	**OR 0.11** (0.04 to 0.36)	**225 fewer per 1,000** (from 249 fewer to 149 fewer)	⨁◯◯◯ Very low[a,b]	IMPORTANT
**Safety (root contact or damage) - Radiographic guide vs Freehand**												
1	randomised trials	serious[c]	not serious	serious[d]	extremely serious[b]	none	3/20 (15.0%)	0/20 (0.0%)	not estimable		⨁◯◯◯ Very low[b,c,d]	IMPORTANT

CI: confidence interval; **OR:** odds ratio

Explanations

a. Includes pre-clinical studies (in vitro, animal, cadaver), which may not directly represent clinical settings. Lack of studies on dynamic Computer-Assisted Surgery (dCAS) reduces the applicability to newer techniques.
b. Limited sample size
c. This study was rated as having "some concerns" in the Risk of Bias Assessement using the Cochrane Collaboration tool.
d. Low external validity

sCAS: static Computer-Assisted Surgery

Table S3. Excluded studies and reasons for exclusion.

Author	Year	Title	Exclusion reason
Al-Suleiman et al.	2011	AUSOM: A 3D placement guide for orthodontic mini-implants	No deviation outcome
Antunes et al.	2017	Three dimensional virtual planning through cone beam computed tomography for surgical guidance production	No miniscrew inserted
Bufalá et al.	2021	Novel digital technique to analyze the influence of the operator experience on the accuracy of the orthodontic micro-screws placement	No guided insertion
Chen et al.	2011	Selection and application of multi-slice CT 3D reconstruction techniques in assisting mini-implant ahchorage implant surgery	No deviation outcome
Choi et al.	2007	A precise wire guide for positioning interradicular miniscrews	Technique description
Chun et al.	2009	The interdental gingiva, a visible guide for placement of mini-implants	No miniscrew inserted
Cui et al.	2022	Effect of a digital guide on the positional accuracy of intermaxillary fixation screw implantation in orthognathic surgery	No deviation outcome
Estelita et al.	2010	Two-dimensional radiographic and clinical references of the tooth crown for orthodontic mini-implant insertion: A guide free technique	No deviation outcome
Kim et al.	2007	Surgical positioning of orthodontic mini-implants with guides fabricated on models replicated with cone-beam computed tomography	Technique description
Kim et al.	2008	Clinical application of a stereolithographic surgical guide for simple positioning of orthodontic mini-implants	Less than 10 miniscrews inserted
Kim et al.	2010	Cone-beam computed tomography evaluation of mini-implants after placement: is root proximity a major risk factor for failure?	No guided insertion
Kirnbauer et al.	2019	Fully guided placement of orthodontic miniscrews - a technical report	No interradicular miniscrew
Landin et al.	2015	A comparative study between currently used methods and small volume-cone beam tomography for surgical placement of mini implants	No deviation outcome
Liu et al.	2019	The effect of a new modified screwdriver in orthodontic	No guided insertion
Ludwig et al.	2022	Accuracy of sterile and non-sterile CAD/CAM insertion guides for orthodontic mini-implants	No interradicular miniscrew
Matzenbacker et al.	2008	The accuracy of radiographic techniques used for vertical localization of mini-implants fixture placement	No miniscrew inserted

Miyazawa et al.	2010	Accurate pre-surgical determination for self-drilling miniscrew implant placement using surgical guides and cone-beam computed tomography	No deviation outcome
Morea et al.	2005	Surgical guide for optimal positioning of mini-implants	Technique description
Paek et al.	2014	Virtually fabricated guide for placement of the C-tube miniplate	Technique description
Paek et al.	2012	A simple customized surgical guide for orthodontic miniplates with tube	Technique description
Präger et al.	2008	Application of a computer navigation system for the placement of orthodontic anchorage screws	No interradicular miniscrew
Riad et al.	2022	Novel digital technique to analyze the accuracy and intraoperative complications of orthodontic self-tapping and self-drilling microscrews placement techniques: An in vitro study	No guided insertion
Rodriguez et al.	2021	Influence of the Computer-Aided Static Navigation Technique on the Accuracy of the Orthodontic Micro-Screws Placement: An In Vitro Study	Duplicated sample
Suzuki et al.	2005	An adjustable surgical guide for miniscrew placement	Technique description
Suzuki et al.	2007	A simple three-dimensional guide for safe miniscrew placement	Technique description
Takahashia et al.	2018	Modified surgical stent for accurate TAD placement	Technique description
Wang et al.	2017	Developing customized dental miniscrew surgical template from thermoplastic polymer material using image superimposition, CAD system, and 3D printing	Less than 10 miniscrews inserted
Wu et al.	2006	Radiographic and surgical template for placement of orthodontic microimplants in interradicular areas: a technical note	No deviation outcome
Yu et al.	2018	Customized surgical template fabrication under biomechanical consideration by integrating CBCT image, CAD system and finite element analysis.	Less than 10 miniscrews inserted

3D: three dimensional; CBCT: cone beam computer tomography; CAD/CAM: computer-aided design and manufacturing; TAD: temporary anchorage devices.
